# Use of a Novel Peptide Welding Technology Platform for the Development of B- and T-Cell Epitope-Based Vaccines

**DOI:** 10.3390/vaccines9050526

**Published:** 2021-05-19

**Authors:** Francesco Nicoli, Salvatore Pacifico, Eleonora Gallerani, Erika Marzola, Valentina Albanese, Valentina Finessi, Sian Llewellyn-Lacey, David A. Price, Victor Appay, Peggy Marconi, Remo Guerrini, Antonella Caputo, Riccardo Gavioli

**Affiliations:** 1 Department of Chemical, Pharmaceutical and Agricultural Sciences, University of Ferrara, 44121 Ferrara, Italy; nclfnc1@unife.it (F.N.); pcfsvt@unife.it (S.P.); glllnr@unife.it (E.G.); mrzrke@unife.it (E.M.); lbnvnt@unife.it (V.A.); fnsvnt@unife.it (V.F.); mcy@unife.it (P.M.); grm@unife.it (R.G.); gvr@unife.it (R.G.); 2 Division of Infection and Immunity, Cardiff University School of Medicine, Cardiff CF14 4XN, UK; Llewellyn-LaceyS@cardiff.ac.uk (S.L.-L.); PriceD6@cardiff.ac.uk (D.A.P.); 3 Systems Immunity Research Institute, Cardiff University School of Medicine, Cardiff CF14 4XN, UK; 4 CNRS UMR 5164, ImmunoConcEpT, Université de Bordeaux, 33000 Bordeaux, France; victor.appay@immuconcept.org

**Keywords:** adaptive immune responses, B-cell epitopes, peptide vaccines, T-cell epitopes

## Abstract

Peptide vaccines incorporating B- and T-cell epitopes have shown promise in the context of various cancers and infections. These vaccines are relatively simple to manufacture, but more immunogenic formulations are considered a priority. We developed tetrabranched derivatives for this purpose based on a novel peptide welding technology (PWT). PWTs provide molecular scaffolds for the efficient synthesis of ultrapure peptide dendrimers, which allow the delivery of multiple ligands within a single macromolecular structure. Peptide vaccines incorporating T-cell epitopes derived from melanoma and B-cell epitopes derived from human immunodeficiency virus, synthesized using this approach, elicited primary immune responses in vitro and in vivo. Subcutaneous administration of the B-cell epitope-based vaccines also elicited more potent humoral responses than subcutaneous administration of the corresponding peptides alone. Highly immunogenic peptide epitope-based vaccines can therefore be generated quickly and easily using a novel PWT.

## 1. Introduction

Immunization is the most effective available strategy for preventing the spread of infectious diseases. However, the eradication of vaccine-preventable infections has been impeded by common misconceptions and uncertainties [1], highlighted by current efforts to immunize the global population against SARS-CoV-2 [2]. The most prevalent concern is safety. These reservations are further compounded in terms of overall coverage by logistical issues related to vaccine storage and transportation. Peptide-based vaccines are attractive in this context, because they are generally safe and have low handling requirements [3,4]. Immunization with linear peptides can be used to elicit specific B- and/or T-cell responses, although the latter approach requires epitope matching to individual human leukocyte antigens (HLAs) [5]. In the setting of personalized medicines for the treatment of cancer, new formulations may also be required periodically to counteract the ongoing emergence of neoantigen specificities, necessitating fast and efficient synthesis methods based on sequence information [6].

The biocompatibility and stability of peptide vaccines render them flexible tools for the local or systemic administration of multiple antigens [3]. However, peptide-based antigens suffer from unfavorable pharmacokinetic properties, including short half-lives in vivo. In addition, the low molecular weight of a peptide antigen may negatively affect its immunogenicity [5,7]. These drawbacks can be overcome using a chemical multimerization strategy to generate immunogenic, peptidase-resistant molecules termed multiple antigen peptides (MAPs) [8]. We recently described a novel approach based on convergent chemistry that allows the rapid synthesis of a new form of MAPs, namely peptide welding technology (PWT) [9]. The tetramerization of different G protein-coupled receptor-derived peptides to PWTs prolonged their action in vivo, potentially as a consequence of reduced susceptibility to the activity of various proteolytic enzymes, including trypsin and chymotrypsin [10,11].

In this study, we present the synthesis of tetrabranched derivatives of three peptide antigens using two cores, PWT1 and PWT2. The PWT1 core is structured on the classical Lys-Lys branched moiety originally developed by Chang et al. [12], whereas the PWT2 core is a cyclam-based structure. B- and T-cell peptide epitopes conjugated to PWT1 or PWT2 were assessed for their immunogenicity in vitro and in vivo. Our results show that the PWT approach allows the efficient delivery of peptides epitopes in a form that elicits primary cellular and humoral immune responses.

## 2. Materials and Methods

### 2.1. Peptides and PWT-Derivatives

Peptides were synthesized using solid-phase Fmoc/tBu chemistry as reported previously [13]. The commercially available Rink amide resin (100 mg, 0.55 mmol/g) was used as a functionalized support for peptides M1, M2, and G1–G4 (Appendix A). The threonine and glutamine Wang resins were used similarly for peptides Tat_1–20_ and Tat_46–60_, respectively. Coupling reactions were conducted for 1 h at room temperature using a 4-fold excess of N,N′-diisopropylcarbodiimide/1-hydroxybenzotriazole (DIC/HOBt). Fmoc removal and further coupling reactions were performed repeatedly to obtain the desired peptide-bound resins. Peptides were removed from the resins via incubation with a TFA/H_2_O/Et_3_SiH mixture (95:2.5:2.5) for 5 h at room temperature. The resulting crude products were triturated with cold diethyl ether, purified using a Waters Prep 600 HPLC System equipped with a Jupiter C18 column (250 × 30 mm, 300 Å, 15 µm particle size), and eluted in a binary mobile phase consisting of solution A (100% H_2_O, 0. 1% *v*/*v* TFA) and solution B (40% H_2_O, 60% CH_3_CN, 0.1% *v*/*v* TFA) at a flow rate of 20 mL/min. Gradients were established individually considering the analytical HPLC profile of the crude product. The molecular weights of the reaction intermediates and final compounds were determined using an electrospray mass spectrometer (ESI MICROMASS ZMD 2000). Purity was assessed using a Beckman Coulter Gold HPLC System 168 equipped with an XBridge C18 column (4.6 × 150 mm, 5 μm particle size) at a flow rate of 0.7 mL/min across a linear gradient from 100% solvent A (H_2_O, 0.1% TFA) to 100% solvent B (CH_3_CN, 0.1% TFA) over 25 min. The analytical profiles of the final compounds (monitored at 220 nm) showed purities > 98%. Resins for solid-phase peptide synthesis and enantiopure Fmoc-protected amino acids were purchased from Bachem (Bubendorf, Switzerland), BLDpharm (Kaiserslautern, Germany), and Fluka (Milan, Italy).

### 2.2. In Vitro Priming of Human Antigen-Specific CD8^+^ T Cells

Buffy coats from healthy blood donors were obtained from the University Hospital of Ferrara. Peripheral blood mononuclear cells (PBMCs) were isolated using Ficoll-Hypaque (Lonza, Basel, Switzerland) and frozen in liquid nitrogen [14,15]. Thawed PBMCs were resuspended at 10^7^ cells/mL in 48-well tissue-culture plates (2.5 × 10^6^ cells/well) containing AIM medium (Thermo Fisher Scientific, Monza, Italy) supplemented with Flt3 ligand (50 ng/mL; Miltenyi Biotec, Bologna, Italy) to mobilize resident dendritic cells. After 24 h (day 1), the Melan-A_26–35_ peptide (ELA) was added to the cultures in its native form or conjugated to PWTs (Appendix A). Negative control wells lacked peptide (PWT scaffold alone). PWT-derivatives were used at a 1:4 molar ratio compared with peptide alone to compensate for stoichiometry (1 µM for ELA and 0.25 µM for PWT2-M1 and PWT2-M2). Dendritic cell maturation was induced with TNF (1000 U/mL; Miltenyi Biotec), IL-1β (10 ng/mL; Miltenyi Biotec), IL-7 (0.5 ng/mL; R&D Systems, Minneapolis, Minnesota, USA), and prostaglandin E2 (1 μM; Calbiochem, Milan, Italy). On day 2, fetal bovine serum (FBS; Euroclone, Milan, Italy) was added at a final *v/v* ratio of 10%. Medium was replaced on days 4 and 7 with fresh RPMI 1640 (Euroclone) enriched with 10% FBS, nonessential amino acids (Euroclone), and sodium pyruvate (Sigma-Aldrich, Milan, Italy). Antigen-specific CD8^+^ T cells were characterized on day 10 as described previously [16,17]. Briefly, cells were labeled with PE-conjugated ELA/HLA-A2 tetramers for 15 min at 37 °C and then surface-stained for 15 min at room temperature with anti-CD3–FITC and anti-CD8–APC-Cy7 (BD Biosciences, Milan, Italy). Intracellular staining for T-bet was performed using a Transcription Factor Buffer Set (BD Pharmingen, Milan, Italy) and anti-T-bet–eFluor 660 (Thermo Fisher Scientific). Non-viable cells were eliminated from the analysis using LIVE/DEAD Fixable Aqua (Thermo Fisher Scientific). Samples were acquired using a FACSCanto II cytometer (BD Biosciences). Data were analyzed using FACSDiva version 7.0 and FlowJo version 10 (BD Biosciences).

### 2.3. Mouse Immunization

Animals (*n* = 5 or 6 per group) were handled according to European and Institutional guidelines. Six-week-old female BALB/c mice (Charles River Laboratories, Lecco, Italy) were immunized subcutaneously (SC) or via the oral mucosa (OM) with the Tat_1–20_ or Tat_46–60_ peptides (3–30 µg), either alone or conjugated to PWT scaffolds (Appendix A). SC injections were performed at two sites on the back with the relevant immunogen diluted in a total of 100 µL of PBS. OM immunization was performed using a pipette tip to deliver the relevant immunogen directly into the mouth in a total of 10 µL of PBS. Mice were deprived of water for 6 h before immunization via this route. Immunogens were administered on days 1, 14, and 28. Serum samples were collected from the retro-orbital plexus 2 or 10 weeks later and processed as described previously [18,19].

### 2.4. Serology

IgG titers specific for the Tat protein from human immunodeficiency virus (HIV) type 1 (isolate IIIB, clone BH10) [15,20] or the Tat_1–20_ or Tat_46–60_ peptides were determined via enzyme-linked immunoassays (ELISAs) as described previously [21,22]. Briefly, 96-well plates were coated with Tat or Tat-derived peptides (100 ng/200 µL/well) in 0.05 M carbonate buffer (pH 9.6) for 18 h at 4 °C. Plates were then washed with PBS containing 0.05% Tween 20 (Sigma-Aldrich) and incubated for 90 min at 37 °C with blocking buffer (PBS containing 0.05% Tween 20 and 1% BSA). After extensive further washes, serial dilutions of each serum sample were dispensed in duplicate wells (100 µL/well) and incubated for 90 min at 37 °C. Plates were washed again before the addition of 100 µL/well of HRP-conjugated goat anti-mouse IgG (Sigma-Aldrich) and then incubated for 90 min at 37 °C. After incubation, plates were washed another five times and developed using a solution (150 µL/well) of 2,2′-azinobis [3-ethylbenzothiazoline-6-sulfonic acid]-diammonium salt (ABTS) substrate (Roche, Monza, Italy). Absorbance values were measured at 405 nm using a Sunrise Absorbance Microplate Reader (Tecan, Salzburg, Austria). Cut-off values were estimated as the mean OD of three negative control sera plus 0.05. Each OD value was subtracted from the blank and cut-off values to obtain a net OD value, and IgG titers were calculated using the intercept function [18,23].

### 2.5. Statistical Analysis

Independent groups were compared using the two-tailed Mann–Whitney U test with Bonferroni correction for multiple comparisons. Time-dependent variations in the number of mice with detectable immune responses were compared using Fisher’s exact test. Significance was assigned at *p* < 0.05.

## 3. Results

### 3.1. Chemistry

**Figure 1:**
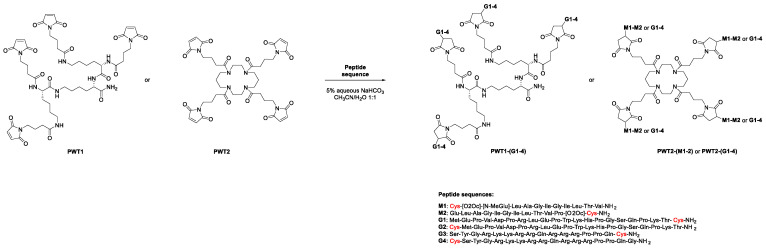
Tetrafunctionalization of PWT1 and PWT2 with the peptide sequences M1–2 and G1–4.

The PWT1 and PWT2 scaffolds were synthesized as described previously [9,24,25,26]. The maleimide derivatives PWT1 and PWT2 were tetrafunctionalized via a thiol-Michael reaction (Figure 1) in the presence of catalytic amounts of a base (NaHCO_3_ 5% aqueous solution) with peptide sequences derived from the melanoma-associated ELA T-cell epitope (M1 and M2) or the HIV-specific Tat_1–20_ (G1 and G2) or Tat_46–60_ (G3 and G4) B-cell epitopes, opportunely modified with a cysteine residue (Appendix A).

Two derivatives of the ELA epitope were synthesized by inserting an N- (M1) or C-terminal (M2) cysteine into the native sequence, and a polyoxyethylene chain was inserted between the peptide sequence and the cysteine residue to increase the solubility of these compounds. Indeed, one of the main practical issues associated with these peptide sequences is that they are highly hydrophobic and insoluble (both in water and organic solvents), with a strong tendency to form gels. Similarly, the N-terminal glutamate residue of M1 was modified to N-methyl glutamate. M1 and M2 were conjugated to PWT2, generating two different constructs [PWT2-(M1–M2)] (Appendix A). This scaffold was used for proof-of-concept immunogenicity studies, because most of the peptide dendrimers generated previously were synthesized using the PWT2 core [9].

The HIV-derived peptides were modified by inserting the cysteine anchoring site at the N- or C-terminal position of Tat_1–20_ (G1 and G2) or Tat_46–60_ (G3 and G4). All four peptides were conjugated separately to PWT1 and PWT2, generating eight different constructs [PWT1-(G1–G4) and PWT2-(G1–G4)] (Appendix A).

Tetrafunctionalization via a thiol-Michael reaction offers the advantage of having an excellent nucleophile under mild reaction conditions (in terms of solvent, temperature, and pH), making it a very efficient and fast procedure (the tetrafunctionalization reaction occurs within 5 min). These features make this chemical strategy applicable to a plethora of peptide sequences and, potentially, also to non-peptide products [27].

### 3.2. PWT Scaffolds Can Present T-Cell Epitopes

In preliminary experiments, we used an in vitro priming system to determine whether HLA class I-restricted peptide epitopes linked to PWT scaffolds could activate naive CD8^+^ T cells [16,28,29]. As a model antigen, we used the HLA-A2-restricted Melan-A_26–35_ peptide epitope (ELA), which is recognized by a relatively large fraction of naive precursors in the human CD8^+^ T-cell pool, facilitating the generation of reproducible data from low numbers of cells [29]. PBMCs from healthy, HLA-A2^+^, melanoma-naive donors were cultured with Flt3 ligand, a standard cocktail of cytokines, and the ELA peptide, either alone or conjugated via its N- (M1) or C-terminus (M2) to PWT2 (Figure 1 and Appendix A). Epitope-specific CD8^+^ T cells were quantified via tetramer staining on day 10.

**Figure 2:**
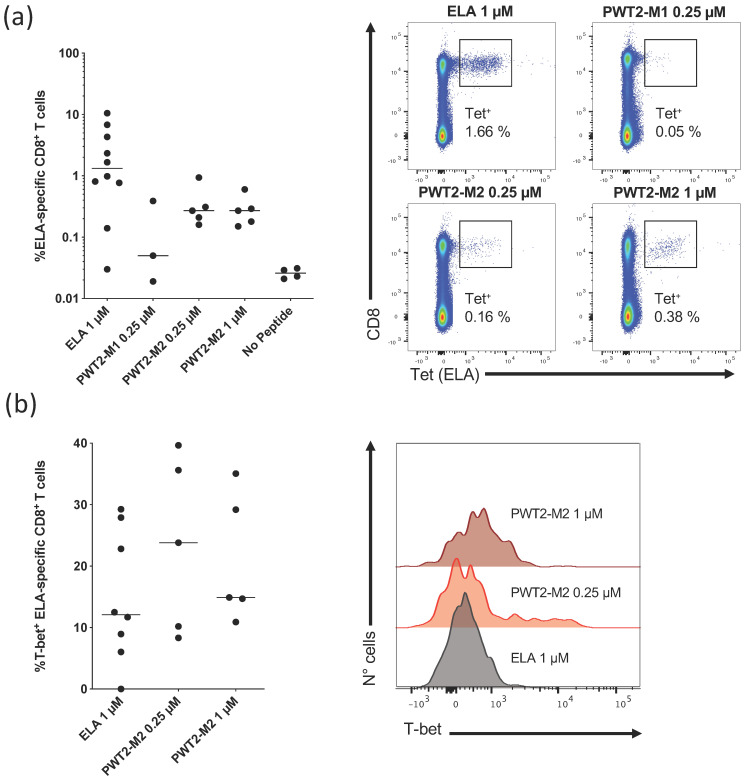
In vitro priming of human naive CD8^+^ T cells specific for a melanoma-associated epitope conjugated to PWT2 (PWT2-M1 and PWT2-M2). (**a**) Data summary (left panel) and representative flow cytometry plots (right panel) showing tetramer^+^ ELA-specific CD8^+^ T cells expanded in the presence of Flt3 ligand, a cocktail of cytokines (TNF, IL-1β, IL-7, and PGE2), and the ELA peptide, either alone or conjugated to PWT2. PBMCs primed in the absence of peptide (PWT2 alone) were used as a negative control. (**b**) Data summary (left panel) and representative flow cytometry histogram plots (right panel) showing intracellular expression of T-bet among the corresponding tetramer^+^ ELA-specific CD8^+^ T cells. Left panels: each dot represents one HLA-A2^+^ donor per condition, and horizontal bars indicate median values (**a**,**b**). Right panels: plots are gated on viable CD3^+^ events (**a**) or on tetramer^+^ ELA-specific CD8^+^ T cells (**b**). Significance was determined using the two-tailed Mann–Whitney U test with Bonferroni correction for multiple comparisons.

PWT2-M1 induced very few ELA-specific CD8^+^ T cells (Figure 2a). Higher response frequencies were observed after priming with PWT2-M2, albeit at levels below those elicited by the cognate peptide alone (Figure 2a). This pattern could not be reversed by increasing the concentration of PWT2-M2 to 1 µM (Figure 2a).

We then evaluated the functionality of ELA-specific CD8^+^ T cells by measuring the expression of T-bet, a master regulator of effector differentiation associated with enhanced cytokine production and cytolytic activity [16,17]. Irrespective of the priming condition, ELA-specific CD8^+^ T cells expressed T-bet at similar frequencies, indicating a consistent pattern of differentiation in vitro (Figure 2b).

Collectively, these results show that the PWT system can present T-cell epitopes in immunogenic form, enabling the differentiation of at least some naive precursors, and that tetrafunctionalization of a cognate peptide does not completely abolish antigen recognition via the TCR.

### 3.3. PWT Scaffolds Can Present B-Cell Epitopes

In further experiments, we used in vivo models to determine whether B-cell epitopes could also elicit immune responses after tetrafunctionalization. Two different PWT scaffolds were used for this purpose: PWT1, which is structurally related to classical MAPs, and PWT2, which is chemically more diverse (Figure 1). As model antigens, we chose two linear B-cell peptides, one immunodominant (Tat_1–20_) and one subdominant (Tat_46–60_), derived from HIV [30]. Each peptide was conjugated separately via the N- and C-terminal domains to PWT1 and PWT2 (Figure 1 and Appendix A). BALB/c mice were injected SC with 3 µg of each construct on days 1, 14, and 28. Control mice were inoculated with the Tat_1–20_ or Tat_46–60_ peptides alone. IgG titers specific for each peptide or the whole Tat protein were measured 2 weeks after the last immunization via ELISA.

As shown in Table 1, the Tat_1–20_ peptide was not immunogenic when administered alone. In contrast, responses were observed in some mice immunized with Tat_1–20_ conjugated to PWT1 via its C-terminal domain (PWT1-G1) or to PWT2 via its N-terminal domain (PWT2-G2). Responses directed against the Tat_1–20_ peptide and the whole Tat protein were observed in both cases. Similarly, the Tat_46–60_ peptide was not very immunogenic when administered alone, inducing a response in only one mouse. Higher levels of immunogenicity were observed with PWT1-G4, PWT2-G3, and PWT2-G4, but not with PWT1-G3. Responses were again specific for both the cognate peptide (Tat_46–60_) and the whole Tat protein.

Collectively, these results show that the PWT system can present B-cell epitopes in immunogenic form, enabling the induction of primary humoral immune responses directed against the cognate peptide and the whole protein antigen.

### 3.4. Conjugation of Peptide Epitopes to PWT Scaffolds Enhances Their Immunogenicity

**Figure 3:**
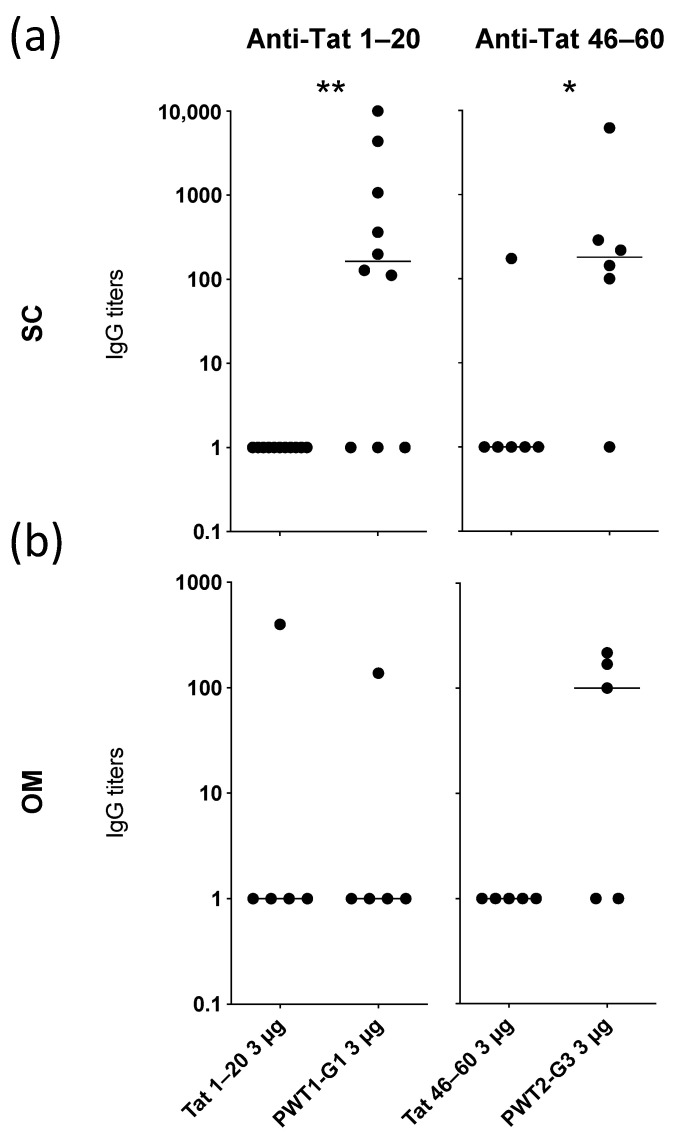
Analysis of IgG responses induced by Tat peptides alone or Tat peptides conjugated to PWT scaffolds. Mice were immunized on three separate occasions with 3 µg of Tat_1–20_, PWT1-G1, Tat_46–60_, or PWT2-G3 delivered either SC (**a**) or via the OM (**b**). Serum samples were collected 2 weeks after the final immunization. IgG titers specific for Tat_1–20_ or Tat_46–60_ were measured via ELISA. Each dot represents one mouse, and horizontal bars indicate median values. Significance was determined using the two-tailed Mann–Whitney U test. * *p* < 0.05, ** *p* < 0.01.

To extend these findings, we focused on the most promising constructs for each specificity, namely PWT1-G1 (Tat_1–20_ conjugated to PWT1 via its C-terminal domain) and PWT2-G3 (Tat_46–60_ conjugated to PWT2 via its N-terminal domain). PWT1-G1 and PWT2-G3 induced significantly higher levels of peptide-specific IgG than the corresponding Tat_1–20_ and Tat_46–60_ peptides alone in mice immunized SC (Figure 3a).

As peptides typically display short half-lives in vivo, with negative consequences for immunogenicity, we immunized mice with the same constructs delivered via the OM. This route of administration exposes antigens to several proteolytic enzymes. Peptides alone (Tat_1–20_ or Tat_46–60_) were poorly immunogenic, and likewise, PWT1-G1 induced Tat_1–20_-specific IgG in only one mouse (Figure 3b). In contrast, OM administration of PWT2-G3 induced Tat_46–60_-specific IgG in 60% of mice (3/5), although this difference was not significant compared with the peptide alone (Figure 3b).

Collectively, these results suggest that humoral immunogenicity can be enhanced by conjugating linear B-cell epitopes to PWT scaffolds, delivered either SC or via the OM.

### 3.5. IgG Titers Induced by Peptides Conjugated to PWT Scaffolds Are Maintained Over Time

**Figure 4:**
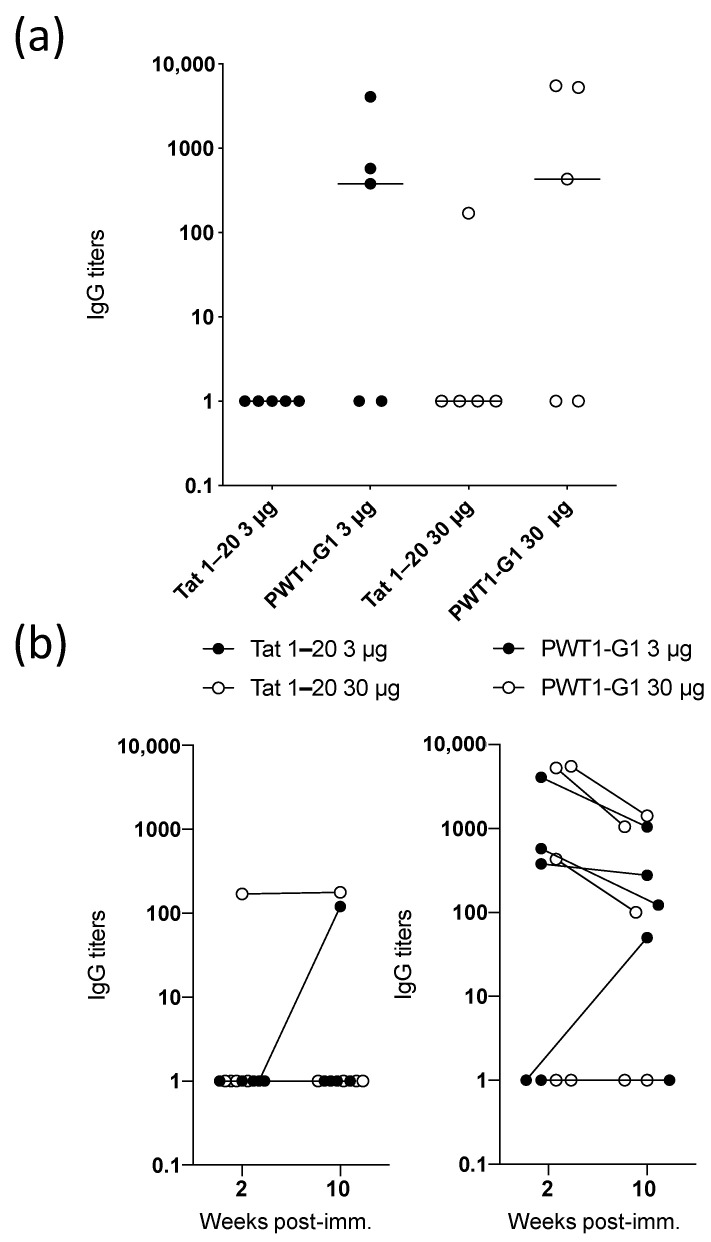
Analysis of IgG responses induced by Tat_1–20_ alone or Tat_1–20_ conjugated to a PWT scaffold over time. (**a**,**b**) Mice were immunized SC on three separate occasions with 3 or 30 µg of Tat_1–20_ or PWT1-G1. Serum samples were collected 2 (**a**,**b**) and 10 weeks (**b**) after the third immunization. IgG titers specific for Tat_1–20_ were measured via ELISA. Each dot represents one mouse, and horizontal bars indicate median values. Significance was determined using the two-tailed Mann–Whitney U test with Bonferroni correction for multiple comparisons (**a**) or Fisher’s exact test (**b**).

Data collected via the in vitro T-cell priming system suggested that increasing the dose of the PWT construct did not affect the magnitude of the subsequent immune response (Figure 2). To explore this phenomenon in the context of B-cell responses, we immunized mice SC with Tat_1–20_ alone or PWT1-G1 at doses of 3 or 30 µg. No significant differences in the resulting IgG titers were observed between the dosing schedules, either for Tat_1–20_ alone or for PWT1-G1 (Figure 4a). Irrespective of dose, however, all mice with detectable IgG responses after 2 weeks also had detectable IgG responses after 10 weeks, indicating that PWT1-G1 induced durable humoral immunity (Figure 4b).

## 4. Discussion

It has been estimated that peptide vaccines are currently being tested for different indications in more than 600 clinical studies, 15 of which have reached phase III [3]. The production of peptide epitopes does not represent a major limitation in this context, because chemical synthesis is rapid and allows the inclusion of modifications at the level of single amino acids, as well as the introduction of linkers and/or stabilizers [3,31]. However, native peptides are susceptible to enzymatic degradation, and more refined approaches are required to enhance the proteolytic stability of clinical-grade products without compromising immunogenicity or purity [3].

**Figure 5:**
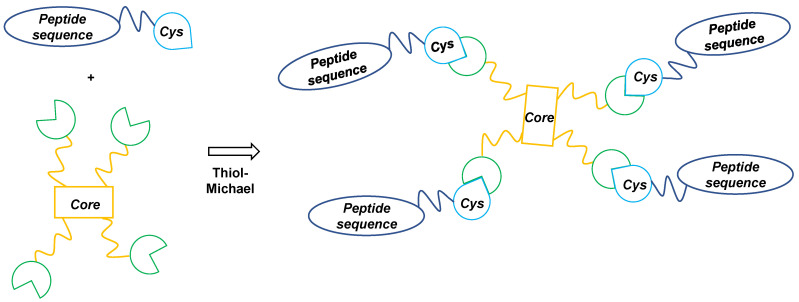
Representation of the convergent chemical strategy.

In this study, we describe a fast, reproducible, and high-yield technology that allows the generation of ultrapure tetrabranched peptides. The classical MAP strategy relies on a solid-phase platform to synthesize branched tetrameric or octameric peptide antigens using an appropriate polylysine-based scaffold [32]. Purification of target compounds prepared via this chemical approach is complex, reflecting contamination by tens of deletion compounds (lacking one or more amino acids) with physicochemical properties (molecular weight, charge, polarity, hydrophilicity, etc.) very similar to those of the desired product. Consequently, overall yields are often very low, with no guarantee of purity [33]. We have developed a convergent chemical strategy for the synthesis of multimeric peptide sequences based on a two-step procedure, in which the peptides and the core are first synthesized and purified separately and then stapled together via the thiol-Michael reaction (Figure 5).

The chemoselectivity of this reaction perfectly matches the need for a highly selective reaction between the core and the peptide binding site. Indeed, the reaction occurs only between the thiol group (characterizing the side-chain of the cysteine residue included in the peptide sequence as Michael’s donor group) and the maleimide function (a typical Michael’s acceptor group) to generate a thioether bond. Using this strategy, both B- and T-cell epitopes could be linked effectively to PWT structures, allowing the induction of primary cellular and humoral immune responses. Moreover, in vivo experiments showed that the levels of epitope-specific IgG induced by peptides conjugated to PWT scaffolds were higher than those elicited by the corresponding native peptides. Of note, not all derivatives were able to prime naive B or T cells. For example, the Tat_1–20_ peptide was not immunogenic when bound to PWT1 via its N-terminus, whereas the Tat_46–60_ peptide was immunogenic in this format. Further studies are necessary to uncover a rational explanation for such discrepancies, which likely relate to the conformation of the final product.

Peptide vaccines have been advocated in the context of various tumors, including melanoma, glioblastoma, and breast cancer [34,35], showing better toxicity profiles than most classical treatments [34]. The first clinical trial of a peptide vaccine was performed in 1995 [36]. More recently, the use of multiple peptides and personalized approaches have shown very promising results in the treatment of melanoma [37,38]. The HLA-A2-restricted, melanoma-associated ELA epitope is among the peptides that have been used in this context [39]. Conjugation of this peptide to a PWT scaffold in the present study allowed the in vitro priming of epitope-specific CD8^+^ T cells. Although the corresponding native peptide was more immunogenic in our system, T-bet expression was induced at similar frequencies among ELA-specific CD8^+^ T cells. In vivo experiments are therefore warranted to pursue these observations and determine whether the greater stability of tetrabranched derivatives could be exploited for clinical applications.

Peptide vaccines have also been advocated for the prevention and treatment of several infectious diseases, including HIV [40,41,42]. For example, the Tat protein has been used in two phase II clinical trials as a therapeutic vaccine against HIV, showing promising results in terms of immunological normalization and viral reservoir reduction [43,44,45]. High levels of antibodies against Tat_1–20_ or Tat_46–60_ have also been associated with undetectable plasma viral loads [46]. In line with this observation, antibodies directed against the N-terminus of Tat, which is the most immunogenic region in terms of humoral responses [47,48], protect monkeys from infection [49]. Moreover, the Tat_46–60_ peptide contains a basic domain that is critical for several Tat-related activities, including transactivation and transportation into the nucleus [50]. In the present study, both peptides were highly immunogenic when conjugated to PWT scaffolds, suggesting that this approach could facilitate the development of vaccines against HIV. In addition, the Tat_46–60_ peptide conjugated to PWT2 elicited more potent humoral immune responses than the corresponding native peptide after mucosal administration. Further studies are required to determine if this effect is a consequence of resistance to proteolysis and in vivo stability. This route of administration nonetheless mimics the anatomy of natural infection and may enhance vaccine compliance as a less invasive mode of immunization [51].

## 5. Conclusions

Our collective data show that PWT scaffolds can present B- and T-cell epitopes. Moreover, humoral immune responses elicited in vivo by peptides conjugated to PWT scaffolds were superior to those elicited in vivo by native peptides and were maintained over time. In conjunction with the advantages of this synthetic approach in terms of rapidity, yield, and purity, these observations suggest that the delivery of peptide vaccines on a global or personalized basis could be enhanced by the widespread adoption of PWT.

## 6. Patents

Remo Guerrini is the inventor of a patent application claiming the PWT technology (EP13162532.9).

## Figures and Tables

**Table 1:** Numbers of mice with detectable humoral responses after immunization.

**Construct**	**PWT**	**Conjugation Domain**	**IgG Responses vs. Tat_1–20_**	**IgG Responses vs. Tat Protein**
Tat_1–20_	Peptide alone	NA ^1^	0/5	0/5
PWT1-G1	PWT1	C-terminus	3/5	2/5
PWT1-G2	PWT1	N-terminus	0/6	0/6
PWT2-G1	PWT2	C-terminus	0/6	0/6
PWT2-G2	PWT2	N-terminus	2/6	3/6
**Construct**	**PWT**	**Conjugation Domain**	**IgG Responses vs. Tat_46–60_**	**IgG Responses vs. Tat Protein**
Tat_46–60_	Peptide alone	NA ^1^	1/6	1/6
PWT1-G3	PWT1	C-terminus	0/6	0/6
PWT1-G4	PWT1	N-terminus	3/6	3/6
PWT2-G3	PWT2	C-terminus	5/6	5/6
PWT2-G4	PWT2	N-terminus	2/6	6/6

^1^ NA, not applicable.

## Data Availability

All data pertaining to this study are included in the article.

## Appendix A

Chemical structures of the compounds used in this study (native peptides and peptides conjugated to PWTs).

ELA peptide sequence:

H-Glu-Leu-Ala-Gly-Ile-Gly-Ile-Leu-Thr-Val-NH_2._

ELA derivative **M1**:

H-Cys-[O2Oc]-[N-Me]Glu-Leu-Ala-Gly-Ile-Gly-Ile-Leu-Thr-Val-NH_2_

ELA derivative **M2**:

H-Glu-Leu-Ala-Gly-Ile-Gly-Ile-Leu-Thr-Val-Pro-[O2Oc]-Cys-NH_2_

Tetrafunctionalized **PWT2** with ELA derivative **M1** (cysteine at N-terminus):

Tetrafunctionalized **PWT2** with ELA derivative **M2** (cysteine at C-terminus):

Tat_1–20_ peptide sequence:

H-Met-Glu-Pro-Val-Asp-Pro-Arg-Leu-Glu-Pro-Trp-Lys-His-Pro-Gly-Ser-Gln-Pro-Lys-Thr-OH

Tat_1–20_ derivative **G1**:

H-Met-Glu-Pro-Val-Asp-Pro-Arg-Leu-Glu-Pro-Trp-Lys-His-Pro-Gly-Ser-Gln-Pro-Lys-Thr-Cys-NH_2_

Tetrafunctionalized **PWT1** with Tat_1–20_ derivative **G1** (cysteine at C-terminus):

Tetrafunctionalized **PWT2** with Tat_1–20_ derivative **G1** (cysteine at C-terminus):

Tat_1–20_ derivative **G2**:

H-Cys-Met-Glu-Pro-Val-Asp-Pro-Arg-Leu-Glu-Pro-Trp-Lys-His-Pro-Gly-Ser-Gln-Pro-Lys-Thr-NH_2_

Tetrafunctionalized **PWT1** with Tat_1–20_ derivative **G2** (cysteine at N-terminus):

Tetrafunctionalized **PWT2** with Tat_1–20_ derivative **G2** (cysteine at N-terminus):

Tat_46–60_ peptide sequence:

H-Ser-Tyr-Gly-Arg-Lys-Lys-Arg-Arg-Gln-Arg-Arg-Arg-Pro-Pro-Gln-OH

Tat_46–60_ derivative **G3**:

H-Ser-Tyr-Gly-Arg-Lys-Lys-Arg-Arg-Gln-Arg-Arg-Arg-Pro-Pro-Gln-Cys-NH_2_

Tetrafunctionalized **PWT1** with Tat_46–60_ derivative **G3** (cysteine at C-terminus):

Tetrafunctionalized **PWT2** with Tat_46–60_ derivative **G3** (cysteine at C-terminus):

Tat_46–60_ derivative **G4**:

H-Cys-Ser-Tyr-Gly-Arg-Lys-Lys-Arg-Arg-Gln-Arg-Arg-Arg-Pro-Pro-Gln-Gly-NH_2_

Tetrafunctionalized **PWT1** with Tat_46–60_ derivative **G4** (cysteine at N-terminus):

Tetrafunctionalized **PWT2** with Tat_46–60_ derivative **G4** (cysteine at N-terminus):
